# Insanity in France

**Published:** 1884-05

**Authors:** 


### INSANITY IN FRANCE.

One of the most striking features of modern French life is the rapid increase of insanity, the number of cases of which, and especially those induced by alcoholism, is becoming larger each year. In 1882 there were 13,434 admissions into the asylums, of which 10,181 were new cases, the total number under treatment in the year being 58,760, of which 27,000 were men, and 31,000 women, showing that females are the most liable to the disease. That they are less influenced by treatment is shown by the fact that the average duration of treatment for men is 276 days, while for women it is 295 days. In France there are 61 public and 42 private asylums, of which 9 are for men only, and 14 for women only, the remainder being for both sexes.

